# Bayesian methods to determine performance differences and to quantify variability among centers in multi-center trials: the IHAST trial

**DOI:** 10.1186/1471-2288-13-5

**Published:** 2013-01-16

**Authors:** Emine O Bayman, Kathryn M Chaloner, Bradley J Hindman, Michael M Todd

**Affiliations:** 1 Department of Anesthesia, The University of Iowa, Iowa City, IA, USA; 2 Department of Biostatistics, The University of Iowa, Iowa City, IA, USA; 3 Department of Statistics & Actuarial Sciences, The University of Iowa, 241 Schaeffer Hall, Iowa City, IA, 52242-1409, USA

**Keywords:** Bayesian outlier detection, Between center variability, Center-specific differences, Exchangeable, Multi-center clinical trial, Performance, Subgroups

## Abstract

**Background:**

To quantify the variability among centers and to identify centers whose performance are potentially outside of normal variability in the primary outcome and to propose a guideline that they are outliers.

**Methods:**

Novel statistical methodology using a Bayesian hierarchical model is used. Bayesian methods for estimation and outlier detection are applied assuming an additive random center effect on the log odds of response: centers are similar but different (exchangeable). The Intraoperative Hypothermia for Aneurysm Surgery Trial (IHAST) is used as an example. Analyses were adjusted for treatment, age, gender, aneurysm location, World Federation of Neurological Surgeons scale, Fisher score and baseline NIH stroke scale scores. Adjustments for differences in center characteristics were also examined. Graphical and numerical summaries of the between-center standard deviation (sd) and variability, as well as the identification of potential outliers are implemented.

**Results:**

In the IHAST, the center-to-center variation in the log odds of favorable outcome at each center is consistent with a normal distribution with posterior sd of *0.538 (95%* credible interval: *0.397* to *0.726*) after adjusting for the effects of important covariates. Outcome differences among centers show no outlying centers. Four potential outlying centers were identified but did not meet the proposed guideline for declaring them as outlying. Center characteristics (number of subjects enrolled from the center, geographical location, learning over time, nitrous oxide, and temporary clipping use) did not predict outcome, but subject and disease characteristics did.

**Conclusions:**

Bayesian hierarchical methods allow for determination of whether outcomes from a specific center differ from others and whether specific clinical practices predict outcome, even when some centers/subgroups have relatively small sample sizes. In the IHAST no outlying centers were found. The estimated variability between centers was moderately large.

## Background

It is important to determine if treatment effects and/or other outcome differences exist among different participating medical centers in multicenter clinical trials. Establishing that certain centers truly perform better or worse than others may provide insight as to why an experimental therapy or intervention was effective in one center but not in another and/or whether a trial’s conclusions may have been impacted by these differences. For multi-center clinical trials, identifying centers performing on the extremes may also explain differences in following the study protocol [1]. Quantifying the variability between centers provides insight even if it cannot be explained by covariates. In addition, in healthcare management, it is important to identify medical centers and/or individual practitioners who have superior or inferior outcomes so that their practices can either be emulated or improved.

Determining whether a specific medical center truly performs better than others can be difficult and/or misleading. Each center enrolls a different patient population, has different standard of care, the sample size varies between centers and is sometimes small. Spiegelhalter recommended using funnel plots to compare institutional performances [2]. Funnel plots are especially useful when sample sizes are variable among centers. When the outcome is binary, the good outcome rates can be plotted against sample size as a measure of precision. In addition, *95%* and *99.8%* exact frequentist confidence intervals are plotted. Centers outside of these confidence bounds are identified as outliers. However, since confidence intervals are very large for small centers, it is almost impossible to detect a center with a small sample size as an outlier or potential outlier using frequentist methods.

Bayesian hierarchical methods can address small sample sizes by combining prior information with the data and making inferences from the combined information. The Bayesian hierarchical model borrows information across centers and thus, accounts appropriately for small sample sizes and leads to different results than the frequentist approach without a hierarchical mixed effects model. A frequentist hierarchical model with components of variance could also be used and also borrows information; however frequentist point estimates of the variance may have large mean square errors compared to Bayesian estimates [3].

The aim of this study is to demonstrate the application of Bayesian methods to determine if outcome differences exist among centers, and if differences in center-specific clinical practices predict outcomes. The variability among centers is also estimated and interpreted. To do so, we utilized data from the Intraoperative Hypothermia for Aneurysm Surgery Trial (IHAST [4]). Specifically, we determined, using a Bayesian mixed effects model, whether outcome variability among IHAST centers was consistent with a normal distribution and/or whether outcome differences can be explained by characteristics of the centers, the patients, and/or specific clinical practices of the various centers.

## Methods

### Frequentist IHAST methods

IHAST was a prospective randomized partially blinded multicenter clinical trial (*1001* subjects, *30* centers) designed to determine whether mild intraoperative systemic hypothermia (*33°C*), compared to normothermia (*36.5°C*), resulted in improved neurologic outcome in subjects with an acute subarachnoid hemorrhage (SAH) undergoing surgery (open craniotomy) to treat a ruptured intracranial aneurysm [4]. A large number of subject and clinical variables were recorded prior to randomization including age, gender, race, World Federation of Neurological Surgeons (WFNS) class, amount of subarachnoid blood (Fisher score), aneurysm size and location, and pre SAH-medical conditions. The details and results of the primary study [4], and subsequent secondary analyses have been previously published [5-9]. The primary outcome measure was the modified Glasgow Outcome Score (GOS) determined 3 months after surgery. The GOS is a five-point functional outcome scale which ranges between 1 (good outcome) and 5 (death) [10]. The primary result of IHAST was that intraoperative hypothermia did not affect neurological outcome: *66% (329 / 499)* good outcome (GOS *= 1*) with hypothermia vs. *63% (314 / 501)* good outcome with normothermia, odds ratio (OR) *= 1.15, 95%* confidence interval: *0.89* to *1.49 *[4].

In IHAST, the randomized treatment assignment (intraoperative hypothermia *vs*. normothermia) was stratified by center such that approximately equal numbers of patients were randomized to hypothermia and normothermia at each participating center. The number of patients contributed by each center ranged between *3* and *93* (median *= 27* patients). A conventional funnel plot showing the proportion of patients with good outcomes by center vs. the number of patients contributed by those centers is implemented.

### Bayesian methods in general

Bayesian inference interprets probability as a degree of belief, and unknown parameters are random variables with prior probability distributions. For example, in IHAST a prior belief was held that the probability of a good outcome would be around *70%* and this probability might range from as low as *30%* in one center and as high as *90%* in another. This information is used to construct the *prior* distribution of the between-center variance. Bayesian methods require that careful attention is paid to the choice of prior distribution [11] and a sensitivity analysis is recommended [12].

The Bayesian approach combines prior information with the clinical trial data and makes inference from this combined information [11,13]. Accordingly, when new clinical trial data become available, the probability distributions are updated, using Bayes theorem, to give a posterior distribution. In contrast, in the traditional approach, probability is interpreted as a long run frequency, giving rise to the terminology “frequentist” inference.

### Bayesian methods applied to the IHAST trial

A Bayesian hierarchical generalized linear model was used for the log odds of a good outcome (defined as a 3-month GOS score of 1). The center effects are additive in the log odds of a good outcome at the different centers and are assumed to be randomly sampled from a normal population; hence they are expected to be different in each center, but similar. In probabilistic terms, this property of “different but similar” is defined as “exchangeable” [14,15]. With the exchangeability assumption, it is assumed *a priori* that good outcome rates for all centers are a sample from the same distribution, and beliefs are invariant to ordering or relabeling of the centers. With the hierarchical model assumption, each center borrows information from the corresponding data of other centers [16]. This is called a shrinkage effect towards the population mean and, as will be shown, this can be especially beneficial when there are small sample sizes in some centers.

As in all prior IHAST publications [5-9], a set of *10* standard covariates were used when exploring the impact of any variable on outcome: preoperative WFNS score (WFNS = 1 or WFNS >1), age (on the continuous scale), gender, Fisher grade on first CT scan, post-SAH National Institute of Health Stroke Scale score (NIHSS), aneurysm location (posterior vs anterior), race, aneurysm size, history of hypertension, and interval from SAH to surgery. These were chosen because of either their demonstrated association with outcome in IHAST or because previous studies had shown them to be associated with outcome following SAH. This set of covariates is included as predictor variables as is treatment assignment (hypothermia vs. normothermia).

In the IHAST *1001* patients were enrolled and randomized, with complete data and follow up is available on *940* subjects. The data set for the *940* subjects is therefore used here.

Let *n*_*jk*_ denote the number of subjects assigned to treatment *j* in center *k* and *X*_*ijk*_ be the values of the covariates for the *i*^*th*^ subject in the *j*^*th*^ treatment group at the *k*^*th*^ center (*i = 1,…,n*_*jk*_*, j = 1,2, k = 1,…,30*). Let *y*_*ijk*_ *= 1* denote a good outcome (GOS = 1) for *i*^*th*^ subject in *j*^*th*^ treatment in center *k and y*_*ijk*_ *= 0* denote GOS >1 for the same subject. Also let $\underline{\beta }$ be the vector of covariates including the intercept *μ* and coefficients *β*_*1*_ to *β*_*11*_ for treatment assignment and the *10* standard covariates given previously. Conditional on the linear predictor ${\underline{x}}_{i}^{T}\underline{\beta }$ and the random center effect *δ*_*k ,*_*y*_*ijk*_ are Bernoulli random variables. Denote the probability of a good outcome, *y*_*ijk*_ = 1, to be *p*_*ijk*_. The random center effects (*δ*_*k,*_*k = 1,…,30*) conditional on the value *σ*_*e*_ are assumed to be a sample from a normal distribution with a mean of zero and sd *σ*_*e .*_ This assumption makes them exchangeable: *δ*_*k*_*| σ*_*e*_ *~ Normal (0, σ*^*2*^_*e*_*).* The value *σ*_*e*_ is the between-center variability on the log odds scale. The point estimate of *σ*_*e*_ is denoted by *s*. The log odds of a good outcome for subject *i* assigned to treatment *j* in center *k* are denoted by *θ*_*ijk*_ *= logit(p*_*ijk*_*) = log(p*_*ijk*_*/(1 – p*_*ijk*_*))* (*i = 1,…,n*_*jk*_*, j = 1,2, k = 1,…,30).*

A model with all potential covariates is $\theta_{\mathrm{ijk}}={\underline{x}}_{i}^{T}\underline{\beta }+\delta_{k}$ and can also be written as follows:

$$\begin{array}{l} \theta_{\mathrm{ijk}}=\mu +\beta_{1}\mathrm{treatmen}t_{j}+\beta_{2}\mathrm{WFN}S_{i}+\beta_{3}\mathrm{ag}e_{i}\\ \;+\beta_{4}\mathrm{gende}r_{i}+\beta_{5}\mathrm{fishe}r_{i}+\beta_{6}\mathrm{strok}e_{i}\\ \;+\beta_{7}\mathrm{locatio}n_{i}+\beta_{8}\mathrm{rac}e_{i}+\beta_{9}\mathrm{siz}e_{i}\\ \;+\beta_{10}\mathrm{hypertensio}n_{i}+\beta_{11}\mathrm{interva}l_{i}+\delta_{k} \end{array}$$

where *μ* is the intercept in the logit scale: *β*_*1*_ to *β*_*11*_ are coefficients to adjust for treatment and *10* standard covariates that are given previously and in Appendix A.1.

Backward model selection is applied to detect important covariates associated with good outcome [17,18]. Covariates are deemed important by checking whether the posterior credible interval of slope term excludes zero. Models are also compared based on their deviance information criteria (DIC) [19]. DIC is a single number describing the consistency of the model to the data. A model with the smaller DIC represents a better fit (see Appendix A.2). Once the important main effects are found, the interaction terms for the important main effects are examined. A model is also fit using all the covariates.

Prior distributions modified from Bayman et al. [20] are used and a sensitivity analysis is performed. Prior distributions for the overall mean and coefficients for the fixed effects are not very informative (see Appendix A.3). The prior distribution of the variance *σ*_*e*_^*2*^ is informative and is specified as an inverse gamma distribution (see Appendix A.3) using the expectations described earlier. Values of *σ*_*e*_ close to zero represent greater homogeneity of centers.

The Bayesian analysis calculates the posterior distribution of the between-center standard deviation, diagnostic probabilities for centers corresponding to “potential outliers”, and graphical diagnostic tools. Posterior point estimates and center- specific *95%* credible intervals (CI) of random center effects (*δ*_*k*_) are calculated. A guideline based on interpretation of a Bayes Factor (*BF*) [14] is proposed for declaring a potential outlier “outlying”. Sensitivity to the prior distribution is also examined [19].

### Specific bayesian methods to determine outlying centers

The method in Chaloner [21] is used to detect outlying random effects. The method extends a method for a fixed effects linear model [22]. The prior probability of at least one center being an outlier is set to *0.05*, and because there are *30* centers, this leads to a definition of an outlying center as one where the magnitude of the random center effect, *δ*_*k*_, is greater than *3.137σ*_*e*_ in absolute value (Appendix A.4)_._ The corresponding prior probability of a specific center being an outlier is 0.0017: Pr(center k is an outlier) = 2 *Φ(−3.137)[22], where *Φ(z)* is the standard normal distribution function.

The posterior probabilities of center *k* being an outlier: *Pr(center k is an outlier | y)* are calculated from the joint posterior distribution of *δ*_*k*_ and *σ*_*e*_[22]. The Bayes factor is also calculated for each of the *30* centers to quantify and interpret the strength of evidence. The *BF* for center *k* is defined as follows:

$$BF_{k}=\frac{\left[1-\mathrm{Pr}\left(\text{center k is an outlier}|y\right)]\mathrm{Pr}(\text{center k is an outlier}\right)}{\mathrm{Pr}\left(\text{center k is an outlier}|y\right)\left[1-\mathrm{Pr}\left(\text{center k is an outlier}\right)\right]}.$$

The *BF* for at least one of the *30* centers being an outlier is also calculated. The proposed method for interpreting the results is that centers where the posterior probability of being an outlier is larger than the prior probability are “potential outliers”. In addition, if *BF*_*k*_ is less than *0.316* then there is “substantial evidence” for center *k* being outlying [14]. Similarly if the *BF* for there being at least one outlying center is less than *0.316* there is substantial evidence for at least one outlying center.

### Bayesian methods regarding other determinants of outcome

In addition to determining if the treatment effect (hypothermia vs. normothermia) differed among any of the *30* IHAST centers and to illustrate our approach on different settings, Bayesian outlier detection methods were applied to determine whether other center-specific subgroups (e.g. number of subjects, geographic location, various clinical practices like nitrous oxide use and temporary clipping) had an effect on outcome (GOS 1 vs. GOS >1).

To determine if the number of subjects enrolled at a center predicted outcome, IHAST centers were categorized *post hoc* by number of enrolled subjects. Let *n*_*k*_ *= n*_*1k*_ *+ n*_*2k*_ and classify centers as either very large (*n*_*k*_ *≥ 69* subjects*; 3* centers*,* 248 subjects), large (*56 ≤ n*_*k*_ *≤ 68* subjects*; 4* centers*, 228* subjects), medium (*31 ≤ n*_*k*_ *≤ 55* subjects*, 7* centers*, 282* subjects*)*) and small (*n*_*k*_ *< 31* subjects*, 16* centers*, 242* subjects). To determine if geographic location predicted outcome, IHAST centers were categorized *post hoc* as being either North American (US and Canada, *22* centers, *637* subjects) or non-North American (Europe, Australia, New Zealand, *8* centers, *363* subjects). To determine if there was evidence of “learning” over the entire course of the study, outcomes of the first *50%* of subjects enrolled in the study (all centers) were compared with outcomes from the second *50%* of subjects enrolled (all centers). Similarly, within each center, the outcomes of first *50%* subjects were compared to the second *50%*.

There are a number of clinical practices which vary among centers that are hypothesized, but not proven, to affect outcome in patients with aneurysmal subarachnoid hemorrhage, such as electrophysiological monitoring, electroencephalography or somatosensory evoked potentials [23], nitrous oxide use [5], temporary clipping [6], *etc*. Centers and/or individual practitioners tend to either embrace these practices (high use) or reject them (low use). Accordingly, Bayesian techniques were used to examine the clinical effect of one anesthetic practice (nitrous oxide use) and one surgical practice (temporary clipping). To determine if the frequency of nitrous oxide use affected outcome, centers were categorized as to their use of nitrous oxide as either low *(≤25%* of the cases, *13* centers), medium (*26%* to *74%* of cases, *8* centers) or high *(≥75%* of cases, *9* centers). In addition, the effect of the nitrous oxide use was explored at the individual subject level (yes, *627* subjects; no, *373* subjects). Finally, the effect of the use of temporary clipping during aneurysm surgery was compared among centers. Centers were categorized as to their frequency of use of temporary clips as low: (*≤30%* of cases; *6* centers), medium: (*30%* to *69%* of cases; *21* centers) and high: (*≥70%* or more of case; *3* centers). The effect of temporary clipping at the individual subject level (yes, *441* subjects; no, *553* subjects) was also examined.

Plots are obtained by R [24], and Bayesian analyses are performed with the WinBUGS [25] program. Model convergence is checked by Brooks, Gelman, Rubin diagnostics plots [26], autocorrelations, density and history plots. A sensitivity analysis is performed.

## Results

### Frequentist analysis

**Figure 1:**
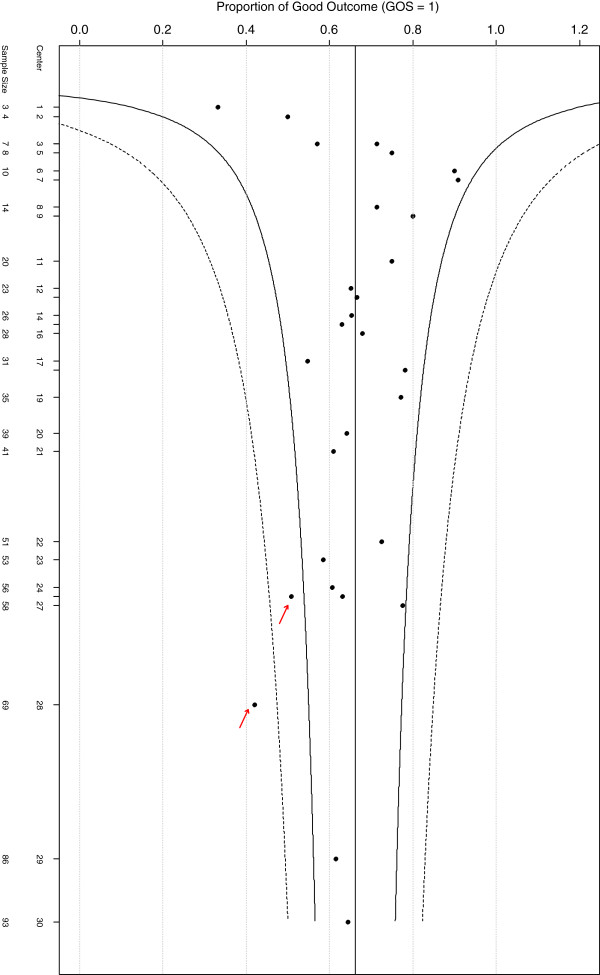
Funnel plot, frequentist, no adjustment for other covariates.

Figure 1 gives the funnel plot [2] for IHAST by center. In this plot, center sizes (*n*_*k*_*)* are plotted against the proportion of good outcome for each center and *95%* and *99.8%* exact binomial confidence intervals are provided. The horizontal line on the funnel plot represents the overall weighted fixed effect good outcome rate (*66%*). Centers outside of the *95%* and *99.8%* confidence bounds are identified as outliers. Accordingly, using this method, IHAST centers *26* and *28* would be identified as outliers, performing less well than the rest of the centers, with good outcome rates of *51%* and *42%*, respectively. However, importantly, patient and center characteristics are not taken into account in this plot.

### Bayesian analysis

A Bayesian hierarchical generalized linear model is fit taking into account the *10* potential covariates and the treatment effect in the model. Covariates are given earlier (see also Appendix A.1). Considering all possible models, the DIC indicates that pre-operative WFNS, Fisher grade on CT scan, pre-operative NIH stroke scale score, aneurysm location (anterior / posterior) and, age should be included in the model. For completeness, gender and treatment are also included as covariates (Appendix A.5). The best model according to DIC adjusts for the main effects of treatment (hypothermia vs. normothermia), WFNS score, gender, Fisher grade on CT scan, pre-operative NIHS stroke scale score, aneurysm location (anterior /posterior), age, center and the interaction of age and pre-operative NIH stroke scale. In this model the log odds of a good outcome for the *i*^*th*^ subject assigned the *j*^*th*^ treatment in center *k* is:

$$\begin{array}{l} \theta_{\mathrm{ijk}}=\mu +\beta_{1}\mathrm{treatmen}t_{j}+\beta_{2}\mathrm{WFN}S_{i}+\beta_{3}\mathrm{ag}e_{i}\\ \;+\beta_{4}\mathrm{gende}r_{i}+\beta_{5}\mathrm{fishe}r_{i}+\beta_{6}\mathrm{strok}e_{i}\\ \;+\beta_{7}\mathrm{locatio}n_{i}+\beta_{8}\mathrm{ag}e_{i}*\mathrm{strok}e_{i}+\delta_{k} \end{array}$$

**Figure 2:**
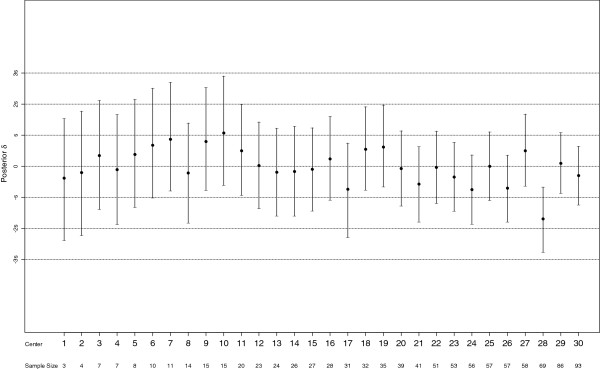
**Posterior mean and *95% *CIs of center log odds of good outcome (*GOS = 1*) for each center are presented under the final model.** Posterior center log odds of good outcome greater than *0* indicates more good outcomes are observed in that center. Horizontal lines show *± s, ±2 s* and *±3 s*, where *s* is the posterior mean of the between-center standard deviation (s = *0.538*, *95%* CI: *0.397* to *0.726*). Centers are ordered by enrollment size.

The model with the posterior means substituted as estimates for the coefficients is:

$$\begin{array}{l} {\hat{\theta }}_{\mathrm{ijk}}=2.024+0.198*\mathrm{treatmen}t_{j}+0.600*\mathrm{WFN}S_{i}\\ \;-0.037*\mathrm{ag}e_{i}-0.256*\mathrm{gende}r_{i}+0.777\\ \;*\mathrm{fishe}r_{i}-0.878*\mathrm{strok}e_{i}-0.788\\ \;*\mathrm{locatio}n_{i}+0.027*\mathrm{ag}e_{i}*\mathrm{strok}e_{i}+\delta_{k} \end{array}$$

and *δ*_*k*_ is the random center effect. The posterior means of the center effects along with *95%* CI’s are given in Figure 2.

There is no evidence of an important treatment effect (hypothermia vs. normothermia). Centers have either greater good outcome rates in both hypothermia and normothermia groups, or lower good outcome rate in both treatment groups (data is not shown). The treatment effect (hypothermia vs. normothermia) within each center was very small. It should be also noted that, when all the potential covariates are included in the model, the conclusions are essentially identical.

In Figure 2 centers are sorted in ascending order of numbers of subjects randomized. For example, *3* subjects were enrolled in center *1* and *93* subjects were enrolled in center *30*. Figure 2 shows the variability between center effects. Consider a 52-year-old (average age) male subject with preoperative WFNS score of *1*, no pre-operative neurologic deficit, pre-operative Fisher grade of *1* and posterior aneurysm. For this subject, posterior estimates of probabilities of good outcome in the hypothermia group ranged from *0.57* (center *28*) to *0.84* (center *10*) across *30* centers under the best model.

The posterior estimate of the between-center sd (*σ*_*e*_) is *s = 0.538* (95% CI of *0.397* to *0.726*) which is moderately large. The horizontal scale in Figure 2 shows *± s, ±2 s* and *±3 s*. Outliers are defined as center effects larger than *3.137σ*_*e*_ and posterior probabilities of being an outlier for each center are calculated. Any center with a posterior probability of being an outlier larger than the prior probability (*0.0017*) would be suspect as a potential outlier. Centers *6*, *7, 10* and *28* meet this criterion; (*0.0020* for center *6, 0.0029* for center *7, 0.0053* for center *10*, and *0.0027* for center *28*). *BF*’s for these four centers are *0.854*, *0.582, 0.323* and *0.624* respectively. Using the *BF* guideline proposed (*BF* < *0.316)* the hypothesis is supported that they are not outliers [14]; all *BF*’s are interpreted as “negligible” evidence for outliers.

The prior probability that at least one of the *30* centers is an outlier is *0.05*. The joint posterior probability that at least one of the *30* centers is an outlier is *0.019*, which is less than the prior probability of *0.05*. Both individual and joint results therefore lead to the conclusion that the no centers are identified as outliers.

Under the normality assumption, the prior probability of any one center to be an outlier is low and is *0.0017* when there are *30* centers. In this case, any center with a posterior probability of being an outlier larger than *0.0017* would be treated as a potential outlier. It is therefore possible to identify a center with a low posterior probability as a “potential outlier”. The Bayes Factor (BF) can be used to quantify whether the relative change from the prior probability of being outlier to the posterior probability is large enough to categorize a center as an outlier.

The use of Bayesian analysis methods demonstrates that, although there is center to center variability, after adjusting for other covariates in the model, none of the *30* IHAST centers performed differently from the other centers more than is expected under the normal distribution. Without adjusting for other covariates, and without the exchangeability assumption, the funnel plot indicated two IHAST centers were outliers. When other covariates are taken into account together with the Bayesian hierarchical model those two centers were not, in fact, identified as outliers. The less favorable outcomes in those two centers were because of differences in patient characteristics (sicker and/or older patients).

### Subgroup analysis

When treatment (hypothermia vs. normothermia), WFNS, age, gender, pre-operative Fisher score, pre-operative NIH stroke scale score, aneurysm location and the interaction of age and pre-operative NIH stroke scale score are in the model and similar analyses for outcome (GOS1 vs. GOS > 1) are performed for four different categories of center size (very large, large, medium, and small) there is no difference among centers—indicating that patient outcomes from centers that enrolled greater numbers of patients were not different than outcomes from centers that enrolled the fewer patients. Our analysis also shows no evidence of a practice or learning effect—the outcomes of the first *50%* of patients did not differ from the outcomes of the second *50%* of patients, either in the trial as a whole or in individual centers. Likewise, an analysis of geography (North American vs. Non-North American centers) showed that outcomes were homogeneous in both locations. The analysis of outcomes among centers as a function of nitrous oxide use (low, medium or high user centers, and on the patient level) and temporary clip use (low, medium, or high user centers and on the patient level) also found that differences were consistent with a normal variability among those strata. This analysis indicates that, overall, differences among centers—either in their size, geography, and their specific clinical practices (e.g. nitrous oxide use, temporary clip use) did not affect patient outcome.

### Sensitivity analysis

**Figure 3:**
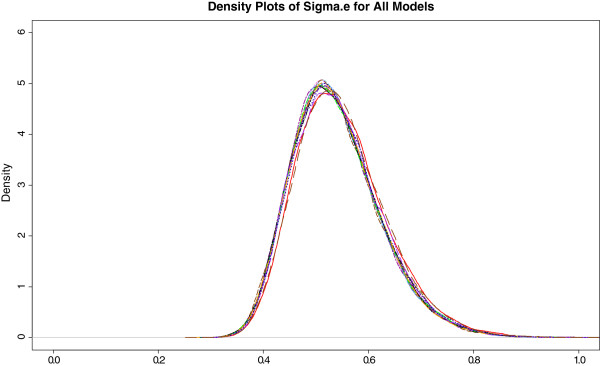
**The posterior density plot of the between-center standard deviation, *σ***_***e*,**_**for *15 *models with variables selected from treatment, age, gender, perioperative WFNS score, baseline NIHHS score, history of hypertension, Fisher grade on CT scan, aneurysm location, aneurysm size, interval from SAH to surgery, and center.**

As a sensitivity analysis, Figure 3 shows the posterior density plots of between-center standard deviation, *σ*_*e*_, for each of *15* models fit. For the first four models, when non important main effects of race, history of hypertension, aneurysm size and interval from SAH to surgery are in the model, *s* is around *0.55*. The point estimate *s* is consistently around *0.54* for the best main effects model and the models including the interaction terms of the important main effects. In conclusion, the variability between centers does not depend much on the covariates that are included in the models.

When other subgroups (center size, order of enrollment, geographical location, nitrous oxide use and temporary clip use) were examined the estimates of between subgroup variability were similarly robust in the corresponding sensitivity analysis.

In summary, the observed variability among centers in IHAST has a moderately large standard deviation (*σ*_*e*_ *= 0.538*, 95% credible interval for *σ*_*e*_*0.397* to *0.726*). No center was declared an outlier and no center-specific or other subgroups were associated with outcome. Sensitivity analyses give similar results.

## Discussion

Although IHAST centers differed in geographic location, experience, and in clinical practices, none of these differences were associated with important differences in outcome. This suggests that although there is moderately large variability among centers, *center-specific variations in patient management* (specifically, nitrous oxide use or temporary clipping) did not greatly affect outcome. If variations in patient management affected outcome, it would be expected that centers with higher enrollment would, as a result of learning, have better outcomes. However, they did not. Likewise, if clinical practices affected outcome, one would expect that outcomes would improve over time as a result of learning. However, our results showed that learning (first *50%* vs last *50%* of subjects to enroll) did not occur and the magnitude of enrollment did not impact outcome. Outcome was however determined in part by patient characteristics such as WFNS, age, pre-operative Fisher score, pre-operative NIHSS stroke scale score, and aneurysm location. Although centers differ in their size, location, and clinical practices, the disease and/or patient characteristics predict patient outcome in this condition.

The greatest advantage of Bayesian techniques over non-hierarchical frequentist methods is its ability to address small sample sizes in some centers. When the stratum-specific sample sizes are small, the hierarchical Bayesian method is especially helpful because information for all centers is averaged with information for a particular center, and weight put on the center specific data proportional to the sample size in the center. Consequently, centers with fewer subjects have less weight put on their center-specific data than do centers with more subjects. Infinite estimates and unbounded confidence intervals arise using only data from subjects in each center to and a frequentist fixed effects model estimate center specific effects, but are avoided using the Bayesian hierarchical model. For example, center *1* enrolled only three subjects: two in the hypothermia group and one in the normothermia group. In the hypothermia group, both patients had an unfavorable outcome, and in the normothermia group the single patient had a good outcome. In this case, the frequentist estimate of the log odds of good outcome for center *1* using only the data from center *1* is infinite and has irregular properties. An alternative practice to avoid infinite estimates is to combine small centers, or to exclude centers with all good outcomes or unfavorable from the analysis [27]. This approach detracts from most preplanned statistical analyses and may reduce the effective sample size. For an intention-to-treat analysis it is essential to include all centers. With the Bayesian approach, and an exchangeability assumption, center estimates are averaged with the overall mean estimate ignoring centers [19]. Extreme center results are therefore systematically adjusted towards the overall average results. As can be seen from Figure 2, the Bayesian estimate of the posterior log odds of good outcome for center *1* uses information from all other centers and has a much narrow range than the frequentist confidence interval. Even if *100%* good outcome rate is observed in center 1, this center is not identified as an outlier center because of the small sample size in this center (*n = 3*). This center does not stand alone and the center-specific estimate borrowed strength from other centers and shifted towards the overall mean.

In the IHAST, two centers (*n*_*26*_ *= 57, n*_*28*_ *= 69*) were identified as outliers by the funnel plot but with the Bayesian approach leading to shrinkage, and also adjustment for covariates they were not declared as outliers. Funnel plots do not adjust for patient characteristics. After adjusting for important covariates and fitting random effect hierarchical Bayesian model no outlying centers were identified.

With the Bayesian approach, small centers are dominated by the overall mean and shrunk towards the overall mean and they are harder to detect as outliers than centers with larger sample sizes. A frequentist mixed model could also potentially be used for a hierarchical model. Bayman et al. [20] shows by simulation that in many cases the Bayesian random effects models with the proposed guideline based on *BF* and posterior probabilities typically has better power to detect outliers than the usual frequentist methods with random effects model but at the expense of the type I error rate.

Prior expectations for variability between centers existed. Not very informative prior distributions for the overall mean, and covariate parameters with an informative distribution on *σ*_*e*_ are used.

The approach proposed in this study is applicable to multiple centers, as well as to any other stratification (group or subgroup) to examine whether outcomes in strata are different. Anesthesia studies are generally conducted in a center with multiple anesthesia providers and with only a few subjects per provider. The approach proposed here can also be used to compare the good outcome rates of anesthesia providers when the outcome is binary (good vs. poor, etc.). This small sample size issue increases the advantage of using Bayesian methods instead of traditional frequentist methods. An additional application of this Bayesian method is to perform a meta-analysis, where the stratification is by study [28].

## Conclusion

The proposed Bayesian outlier detection method in the mixed effects model adjusts appropriately for sample size in each center and other important covariates. Although there were differences among IHAST centers, these differences are consistent with the random variability of a normal distribution with a moderately large standard deviation and no outliers were identified. In addition, no evidence was found for any known center characteristic to explain the variability. This methodology could prove useful for other between-centers or between-individuals comparisons, either for the assessment of clinical trials or as a component of comparative-effectiveness research.

## Statistical appendix

### A.1. List of potential covariates

The potential covariates and their definitions are: treatment (hypothermia vs normothermia), preoperative WFNS score(*1* vs *>1*), age, gender, race (white vs others), Fisher grade on CT scan (*1* vs others), pre-operative NIHS stroke scale score (*0* vs others), aneurysm location (posterior vs anterior), aneurysm size (largest diameter of first aneurysm *≥ 25* vs *<25*), history of hypertension (yes vs no) and interval from SAH to surgery (*0* to *7* days vs *8* to *14* days).

### A. 2. Deviance Information Criterion (DIC)

The expected predicted deviance is suggested as a measure of model comparison and adequacy to compare the fit of different models to the same data [18,19]. The deviance information criterion *(DIC)* is the difference between the estimated average discrepancy and the discrepancy of the point estimate and is a single number. The model with a smaller DIC value is preferred to the model with a larger DIC.

### A. 3. Justification and Description of Prior Distributions

**Figure 4:**
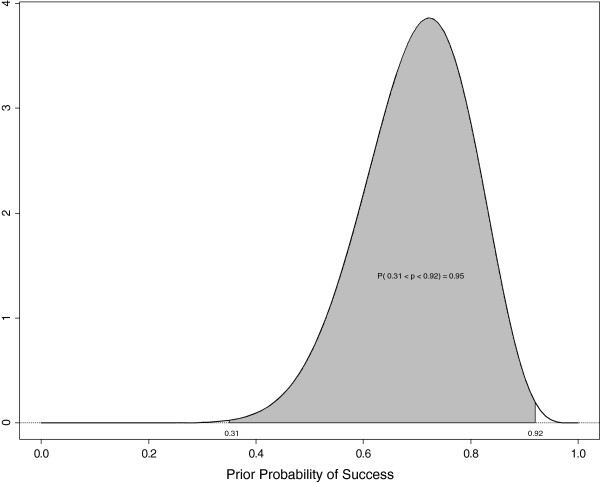
**The probability density function for *σ***_***e***_**and *95% *prior probability interval.**

Prior distributions for covariatesPrior distributions for the overall mean (*μ*), main effects of treatment, coefficient corresponding to preoperative WFNS score, gender, race, Fisher grade on CT scan, pre-operative NIHS stroke scale score, aneurysm location, aneurysm size, history of hypertension and interval from SAH to surgery are assumed to be a normal distribution with mean zero and standard deviation *10*. This distribution is not very informative. Because age is measured in years, and has a wider scale, the prior distribution for the regression coefficient of age at randomization is a normal distribution centered zero with standard deviation *1*. Similarly, the prior distribution for the coefficient corresponding to interaction of age by any other covariate is normally distributed with mean zero and a standard deviation of *1*.As explained in the *Bayesian Methods Applied to the IHAST Trial section, the* prior distribution for the between-center variance $\left(\underset{e}{\sigma^{2}}\right)$ is assumed to be an inverse gamma distribution with mean *0.667* and standard deviation *0.471*. For this Inverse Gamma distribution*,* the prior probability is *95%* that any center’s log odds of a good outcome lies between *31%* and *92%*. This prior probability distribution is illustrated in Figure 4.

### A. 4. Calculating the Prior Probability of Being an Outlier

An outlier can be defined based on specifying the prior probability of not having any outliers as very high, say *95%*. Then the prior probability of a specific center *k* being an outlier when there are *n* centers is *2Φ(−m)* where *m = Φ*^*-1*^[0.5 + ½ * (0.95^*1/n*^*)]*[22]. For example, when comparing *30* centers, *n = 30 and m* is *3.137* and the prior probability of being outlier for a specific center is *0.0017*.

### A. 5. Treatment and Gender as Covariates in the Final Model

In the model selection process using the DIC criterion, treatment effect is not an important covariate. However, given that in IHAST subjects are randomized to treatment, hypothermia or normothermia, this covariate is included in the final model. Similarly, according to DIC criterion gender is not an important covariate, however as the interaction between gender and treatment effect is deemed important it is included.

### A. 6. Appendix

The members of IHAST were as follows: **University of Iowa — Steering Committee:** M. Todd, B. Hindman, W. Clarke, K. Chaloner, J. Torner, P. Davis, M. Howard, D. Tranel, S. Anderson; **Clinical Coordinating Center:** M. Todd, B. Hindman, J. Weeks, L. Moss, J. Winn; **Data Management Center:** W. Clarke, K. Chaloner, M. Wichman, R. Peters, M. Hansen, D. Anderson, J. Lang, B. Yoo; **Physician Safety Monitor:** H. Adams; **Project Advisory Committee** — G. Clifton (University of Texas, Houston), A. Gelb (University of California, San Francisco), C. Loftus (Temple University, Philadelphia), A. Schubert (Cleveland Clinic, Cleveland); **Physician Protocol Monitor** — D. Warner (Duke University, Durham, N.C.); **Data and Safety Monitoring Board** — W. Young, chair (University of California, San Francisco), R. Frankowski (University of Texas Health Science Center at Houston School of Public Health, Houston), K. Kieburtz (University of Rochester School of Medicine and Dentistry, Rochester, N.Y.), D. Prough, University of Texas Medical Branch, Galveston), L. Sternau (Mt. Sinai Medical Center, Miami); **NIH, National Institute of Neurological Disease and Stroke, Bethesda, Md. —** J. Marler, C. Moy, B. Radziszewska; **Participating Centers (the number of randomized patients at each center is listed in parentheses): Addenbrooke’s Hospital, Cambridge, United Kingdom (93):** B. Matta, P. Kirkpatrick, D. Chatfield, C. Skilbeck, R. Kirollos, F. Rasulo, K. English, C. Duffy, K. Pedersen, N. Scurrah, R. Burnstein, A. Prabhu, C. Salmond, A. Blackwell, J. Birrell, S. Jackson; **University of Virginia Health System, Charlottesville (86):** N. Kassell, T. Pajewski, H. Fraley, A. Morris, T. Alden, M. Shaffrey, D. Bogdonoff, M. Durieux, Z. Zuo, K. Littlewood, E. Nemergut, R. Bedford, D. Stone, P. Balestrieri, J. Mason, G. Henry, P. Ting, J. Shafer, T. Blount, L. Kim, A. James, E. Farace, L. Clark, M. Irons, T. Sasaki, K. Webb; **Auckland City Hospital, Auckland, New Zealand (69):** T. Short, E. Mee, J. Ormrod, J. Jane, T. Alden, P. Heppner, S. Olson, D. Ellegala, C. Lind, J. Sheehan, M. Woodfield, A. Law, M. Harrison, P. Davies, D. Campbell, N. Robertson, R. Fry, D. Sage, S. Laurent, C. Bradfield, K. Pedersen, K. Smith, Y. Young, C. Chambers, B. Hodkinson, J. Biddulph, L. Jensen, J. Ogden, Z. Thayer, F. Lee, S. Crump, J. Quaedackers, A. Wray, V. Roelfsema; **Sozialmedizinisches Zentrum Ost–Donauspital, Vienna (58):** R. Greif, G. Kleinpeter, C. Lothaller, E. Knosp, W. Pfisterer, R. Schatzer, C. Salem, W. Kutalek, E. Tuerkkan, L. Koller, T. Weber, A. Buchmann, C. Merhaut, M. Graf, B. Rapf; **Harborview Medical Center, Seattle (58):** A. Lam, D. Newell, P. Tanzi, L. Lee, K. Domino, M. Vavilala, J. Bramhall, M. Souter, G. Britz, H. Winn, H. Bybee; **St. Vincent’s Public Hospital, Melbourne, Australia (57):** T. Costello, M. Murphy, K. Harris, C. Thien, D. Nye, T. Han, P. McNeill, B. O'Brien, J. Cormack, A. Wyss, R. Grauer, R. Popovic, S. Jones, R. Deam, G. Heard, R. Watson, L. Evered, F. Bardenhagen, C. Meade, J. Haartsen, J. Kruger, M. Wilson; **Universityof Iowa Health Care, Iowa City (56):** M. Maktabi, V. Traynelis, A. McAllister, P. Leonard, B. Hindman, J. Brian, F. Mensink, R. From, D. Papworth, P. Schmid, D. Dehring, M. Howard, P. Hitchon, J. VanGilder, J. Weeks, L. Moss, K. Manzel, S. Anderson, R. Tack, D. Taggard, P. Lennarson, M. Menhusen; **University of Western Ontario, London, Ont., Canada (53):** A. Gelb, S. Lownie, R. Craen, T. Novick, G. Ferguson, N. Duggal, J. Findlay, W. Ng, D. Cowie, N. Badner, I. Herrick, H. Smith, G. Heard, R. Peterson, J. Howell, L. Lindsey, L. Carriere, M. von Lewinski, B. Schaefer, D. Bisnaire, P. Doyle-Pettypiece, M. McTaggart; **Keck School of Medicine at University of Southern California, Los Angeles (51):** S. Giannotta, V. Zelman, E. Thomson, E. Babayan, C. McCleary, D. Fishback; **University of Michigan Medical Center, Ann Arbor (41):** S. Samra, B. Thompson, W. Chandler, J. Mcgillicuddy, K. Tremper, C. Turner, P. Smythe, E. Dy, S. Pai, V. Portman, J. Palmisano, D. Auer, M. Quigley, B. Giordani, A. Freymuth, P. Scott, R. Silbergleit, S. Hickenbottom; **University of California San Francisco, San Francisco (39):** L. Litt, M. Lawton, L. Hannegan, D. Gupta, P. Bickler, B. Dodson, P. Talke, I. Rampil, B. Chen, P. Wright, J. Mitchell, S. Ryan, J. Walker, N. Quinnine, C. Applebury; **Alfred Hospital, Melbourne, Australia (35):** P. Myles, J. Rosenfeld, J. Hunt, S. Wallace, P. D’Urso, C. Thien, J. McMahon, S. Wadanamby, K. Siu, G. Malham, J. Laidlaw, S. Salerno, S. Alatakis, H. Madder, S. Cairo, A. Konstantatos, J. Smart, D. Lindholm, D. Bain, H. Machlin, J. Moloney, M. Buckland, A. Silvers, G. Downey, A. Molnar, M. Langley, D. McIlroy, D. Daly, P. Bennett, L. Forlano, R. Testa, W. Burnett, F. Johnson, M. Angliss, H. Fletcher; **Toronto Western Hospital, University Health Network, Toronto (32):** P. Manninen, M. Wallace, K. Lukitto, M. Tymianski, P. Porter, F. Gentili, H. El-Beheiry, M. Mosa, P. Mak, M. Balki, S. Shaikh, R. Sawyer, K. Quader, R. Chelliah, P. Berklayd, N. Merah, G. Ghazali, M. McAndrews, J. Ridgley, O. Odukoya, S. Yantha; **Wake Forest University Baptist Medical Center, Winston-Salem, N.C. (31):** J. Wilson, P. Petrozza, C. Miller, K. O’Brien, C. Tong, M. Olympio, J. Reynolds, D. Colonna, S. Glazier, S. Nobles, D. Hill, H. Hulbert, W. Jenkins; **Mayo Clinic College of Medicine, Rochester, Minn. (28):** W. Lanier, D. Piepgras, R. Wilson, F. Meyer, J. Atkinson, M. Link, M. Weglinski, K. Berge, D. McGregor, M. Trenerry, G. Smith, J. Walkes, M. Felmlee-Devine; **West fälische Wilhelms-Universitat Muenster, Muenster, Germany (27):** H. Van Aken, C. Greiner, H. Freise, H. Brors, K. Hahnenkamp, N. Monteiro de Oliveira, C. Schul, D. Moskopp, J. Woelfer, C. Hoenemann, H. Gramke, H. Bone, I. Gibmeier, S. Wirtz, H. Lohmann, J. Freyhoff, B. Bauer; **University of Wisconsin Clinical Science Center, Madison (26):** K. Hogan, R. Dempsey, D. Rusy, B. Badie, B. Iskandar, D. Resnick, P. Deshmukh, J. Fitzpatrick, F. Sasse, T. Broderick, K. Willmann, L. Connery, J. Kish, C. Weasler, N. Page, B. Hermann, J. Jones, D. Dulli, H. Stanko, M. Geraghty, R. Elbe; **Montreal Neurological Hospital, Montreal (24):** F. Salevsky, R. Leblanc, N. Lapointe, H. Macgregor, D. Sinclair, D. Sirhan, M. Maleki, M. Abou-Madi, D. Chartrand, M. Angle, D. Milovan, Y. Painchaud; **Johns Hopkins Medical Institutions, Baltimore (23):** M. Mirski, R. Tamargo, S. Rice, A. Olivi, D. Kim, D. Rigamonti, N. Naff, M. Hemstreet, L. Berkow, P. Chery, J. Ulatowski, L. Moore, T. Cunningham, N. McBee, T. Hartman, J. Heidler, A. Hillis, E. Tuffiash, C. Chase, A. Kane, D. Greene-Chandos, M. Torbey, W. Ziai, K. Lane, A. Bhardwaj, N. Subhas; **Cleveland Clinic Foundation, Cleveland (20):** A. Schubert, M. Mayberg, M. Beven, P. Rasmussen, H. Woo, S. Bhatia, Z. Ebrahim, M. Lotto, F. Vasarhelyi, J. Munis, K. Graves, J. Woletz, G. Chelune, S. Samples, J. Evans, D. Blair, A. Abou-Chebl, F. Shutway, D. Manke, C. Beven; **New York Presbyterian Hospital–Weill Medical College of Cornell University, New York (15):** P. Fogarty-Mack, P. Stieg, R. Eliazo, P. Li, H. Riina, C. Lien, L. Ravdin, J. Wang, Y. Kuo; **Stanford University Medical Center, Palo Alto, Calif. (15):** R. Jaffe, G. Steinberg, D. Luu, S. Chang, R. Giffard, H. Lemmens, R. Morgan, A. Mathur, M. Angst, A. Meyer, H. Yi, P. Karzmark, T. Bell-Stephens, M. Marcellus; **Plymouth Hospitals National Health Service Trust, Plymouth, United Kingdom (14):** J. Sneyd, L. Pobereskin, S. Salsbury, P. Whitfield, R. Sawyer, A. Dashfield, R. Struthers, P. Davies, A. Rushton, V. Petty, S. Harding, E. Richardson; **University of Pittsburgh Medical Center, Pittsburgh (11):** H. Yonas, F. Gyulai, L. Kirby, A. Kassam, N. Bircher, L. Meng, J. Krugh, G. Seever, R. Hendrickson, J. Gebel; **Austin Health, Melbourne, Australia (10):** D. Cowie, G. Fabinyi, S. Poustie, G. Davis, A. Drnda, D. Chandrasekara, J. Sturm, T. Phan, A. Shelton, M. Clausen, S. Micallef; **Methodist University Hospital, Memphis, Tenn. (8):** A. Sills, F. Steinman, P. Sutton, J. Sanders, D. Van Alstine, D. Leggett, E. Cunningham, W. Hamm, B. Frankel, J. Sorenson, L. Atkins, A. Redmond, S. Dalrymple; **University of Alabama at Birmingham, Birmingham (7):** S. Black, W. Fisher, C. Hall, D. Wilhite, T. Moore II, P. Blanton, Z. Sha; **University of Texas Houston Health Science Center, Houston (7):** P. Szmuk, D. Kim, A. Ashtari, C. Hagberg, M. Matuszczak, A. Shahen, O. Moise, D. Novy, R. Govindaraj; **University of Colorado Health Science Center, Denver (4):** L. Jameson, R. Breeze, I. Awad, R. Mattison, T. Anderson, L. Salvia, M. Mosier; **University of Oklahoma Health Science Center, Oklahoma City (3):** C. Loftus, J. Smith, W. Lilley, B. White, M. Lenaerts.

## Competing interests

The authors declare that they have no competing interests.

## Authors’ contributions

EOB Conception and design, analysis and interpretation of data, drafting the article and revising it critically for important intellectual content. KMC Conception and design, interpretation of data, revising the article critically for important intellectual content and final approval of the version to be published. BJH Revising the article critically for important intellectual content and final approval of the version to be published. MMT Principal Investigator of the IHAST trial, intellectual contributor to the development and evolution of the article, and actively involved in reviewing/revising the article for content as well as providing final approval for the version to be published. All authors have read and approved the final manuscript.

## Pre-publication history

The pre-publication history for this paper can be accessed here:

http://www.biomedcentral.com/1471-2288/13/5/prepub
